# Parental alienation in Lebanon: a case report

**DOI:** 10.1186/s13256-023-03911-3

**Published:** 2023-04-23

**Authors:** Rabih Fares, Roudna Najem, Souheil Hallit, Antoine Pelissolo, Georges Haddad, Wadih J. Naja

**Affiliations:** 1 Departments of Psychiatry and Research, Psychiatric Hospital of the Cross, Jal Eddib, Lebanon; 2 School of Medicine and Medical Sciences, Holy Spirit University of Kaslik, P.O. Box 446, Jounieh, Lebanon; 3 Clinical Psychologist/Psychotherapist, Private Clinic, Beirut, Lebanon; 4 Applied Science Research Center, Applied Science Private University, Amman, Jordan; 5 IMRB Translational Neuropsychiatry Laboratory, Department of Psychiatry, AP-HP, Hopital Henri-Mondor, University Paris Est Creteil, 94010 Creteil, France; 6 Department of Psychiatry, Faculty of Medicine, Lebanese University, Hadat, Lebanon

**Keywords:** Parental alienation, Alienating parent, Alienated parent, Child custody disputes, Case report

## Abstract

**Background:**

Parental alienation is a relatively newly described disorder, with a growing prevalence, as divorce and custody battles are becoming more and more complex with increased difficulty of joint custody. In parental alienation, one parent, the alienating parent, forms an alliance with the child involved in the custody dispute and manages to effectively alienate the targeted parent completely. The child and the alienating parent manifest a form of *folie à deux* and, hence, are in complete synchrony in the hatred and denigration of the targeted parent. Issues, such as potentially false allegations of sexual, physical, and emotional abuse of the child by the targeted parent, arise. The child and the alienating parent become mutually convinced of the targeted parent’s transgressions. Consequently, it becomes difficult for the courts and psychiatric professionals to differentiate true abuse from parental alienation.

**Case presentation:**

In this case study, we aimed to conduct an in-depth psychological and psychiatric evaluation of a Lebanese family (white race) where a father was wrongly accused by the mother and his 11-year-old white boy of both physical and sexual abuse. The data for this study were collected through unstructured and semi-structured interviews, observations, and psychological tests (Rorschach test for the parents and Blacky test for the child), and through the analysis of documented evidence presented in the trial.

**Conclusion:**

This case manifested most criteria set forth for the diagnosis of parental alienation and created serious doubt regarding the validity of the allegations set forth by the mother and the child. Uncovered parental alienation often misleads mental health professionals at the expense of the child’s mental health .

## Background

In the early 1970’s, a new law was established in Michigan to protect the best interest of the child and grant minor children the rights of a relationship with both parents during a custody dispute. Thus, the “tender years presumption,” which assumed that the mother knows best, was replaced by the “best interest of the child” presumption, which is based on the idea that the best parent is “both parents” [1]. This law has led to custodial arrangements that benefit the child by ensuring the receipt of emotional (love, affection, and so on) and basic life needs (food, clothing, medical care, and so on) from both parents. However, this is not always the case; custodial arrangements could have more precarious effects when parents are at war over the custody of a child. As a result, one parent may try to damage the relationship of the child with the other parent and turn the child’s emotion against that other parent.

By the 1980’s, Dr. Richard Gardner, a forensic psychiatrist, began to observe a previously and rarely described disorder in which the child is being turned against one parent in extended custody litigation [2, 3]. He was the first to identify and name this phenomenon as parental alienation syndrome (PAS). Later, during 1986, two psychologists in Michigan, Blush and Ross, who were unaware of the publications and research of Gardner, published the first of several papers on sex abuse allegations in divorce (SAID) syndrome [4]. They outlined typologies for the accused parent, the child involved, and the falsely accusing parent, the latter parent frequently being the mother. Subsequently, Jacobs in New York and Wallerstein in California published case reports of what they called Medea syndrome [5, 6], mothers who took their revenge on their former husbands by depriving them of their children. Jacobs commented on Gardner’s work on PAS in his study (1988) of a mother with Medea syndrome, a severe form of alienation. So did Turkat when he described divorce-related malicious mother syndrome, where the divorced or divorcing parent tends to harm the child so that the other parent may look bad, as an abnormal behavior during divorce [7]. It must be noted that fathers too have been described to have this disorder [8].

Throughout the years, many authors went on to omit the term “syndrome,” describing parental alienation (PA) as a disorder arising primarily in the context of child custody disputes [9]. However, PA does not exist in the International Classification of Diseases (ICD-11) or in the Diagnostic and Statistical Manual of Mental Disorders, 5th edition, text revision (DSM-5-TR). The main reason behind its non-classification is the lack of studies in the scientific literature that strengthen the validity of this concept. Subsequently, many definitions of parental alienation have been offered; however, the most compelling definition is still the one from Dr. Richard Gardner since it includes the causes of PA. As per Gardner, PA is primarily manifested as the preoccupation of the child with the deprecation and criticism of one parent, where the denigration of the targeted parent is unjustified and/or exaggerated [10]. The campaign of condemnation of the parent results from a combination of brainwashing or programming of the child by the accusing parent and the child’s own contribution to the slander of the targeted parent. These two factors may, and usually do, mutually reinforce one another.

As a consequence, Harman showed in his review that PA is still socially and politically denied and highlighted the need for better diagnostic tools to recognize and intervene early to benefit the alienating child [11]. Based on their understanding of the components of PA, Bernet and Greenhill established the Five-Factor Model (FFM) to aid in diagnosis. These factors consist of (1) the child’s avoidance of one of the parents; (2) the presence of a prior positive relationship between the child and the alienated parent; (3) the absence of abuse, deficient parenting, or neglect by the alienated parent; (4) the use of multiple alienating behaviors on the part of the alienating parent; and (5) the child’s exhibition of many alienating behavioral signs [12].

Families affected by PA experience some degree of trauma. The alienating child lives in what is essentially a cult, whose main objective is the alienation of the targeted parent. Therefore, those who are charged with the investigation of allegations of child abuse must be very sensitive to the situation and always consider the best interest of the child, without causing damage to potentially innocent families or parents against whom accusations are made.

The scientific literature suggests that PA is a real phenomenon that affects the mental health of thousands of children and their families. However, PA is not recognized in many countries including Great Britain, and evidence-bases interventions specifically for PA do not exist. Moreover, there is not enough published research to support the inclusion of PA in the DSM 5-TR or the ICD-11. Our aim is to contribute to the scientific literature on PA and highlight the importance of early recognition by showing its psychological repercussion. Only after PA is recognized can proper treatment take place.

Our manuscript is the first Lebanese case report of parental alienation. We conducted an in-depth psychological and psychiatric investigation of a case where the father was accused by the mother and the child of physical and sexual abuse of the latter. The father claimed that he was wrongly accused of this crime. We will also speak about the consequences suffered by the accused parent while trying to illustrate the measures that were taken to ensure the best interest of the child.

## Case presentation

Data were collected through unstructured and semi-structured interviews, observations, psychological tests (Rorschach test for the parents and Blacky test for the child), and analysis of documented evidence that was presented in the trial. Written consent was obtained from the parents.

### The accused parent’s account of the events

Mr. R is the second of a family of five children who had a calm and pleasant childhood and adolescence. He met Mrs. R, his wife, who supported him leaving to go to Argentina in 2000 to work as a courier following the economic difficulties in Lebanon. They got married in 2003 in Turkey. The husband reported that the wife spent the honeymoon in tears, as she was very upset about leaving her family. He stated that he supported his wife during this phase since she was an only child and very attached to her parents. A few days after, Mr. and Mrs. R moved to Argentina together.

Mr. R. was a hard worker and reached the position of operation manager in his company. Four years later, during Mrs. R’s fifth month of pregnancy, she decided to leave her husband in Argentina and come back to Lebanon to deliver their first son, J. However, she refused to rejoin her husband in Argentina after her delivery, claiming that he had mistreated her and that she wanted to stay in Lebanon. Conflicts started between the couple; as a result, Mr. R decided to file for a divorce. Following this decision, Mrs. R allowed her husband to listen to J’s breathing and crying through phone calls. However, when he refused to return to Lebanon, she forbade him from speaking with the child. She even took legal actions to prohibit Mr. R from seeing his son in case he decided to return to Lebanon. During the 3-year divorce process, she refused to give Mr. R the right to speak to his son, until he was able to secure a court order of his own, allowing him to return and see his son. Despite the court order, the mother refused to give Mr. R the right to see the child until the court imposed a term of imprisonment if she refused to comply with the court order. Therefore, Mr. R’s first meeting with J was held in the house of a relative of the child’s mother when J was 3 years old.

Three years later, in 2014, Mrs. R suggested bringing J to Argentina for a 2-week vacation with his father. The vacation was an outstanding success, and the bond between J and his father strengthened. In 2016, Mrs. R proposed returning to live in Argentina with Mr. R, who accepted for the sake of the child. However, soon after the reunion of the family, Mr. R noticed that Mrs. R was frequently using a nonverbal language with J that he could not understand, which made him feel excluded. As a result, the relationship between the father and his son began to deteriorate.

After spending a year in Argentina, the family decided to visit Lebanon for a summer vacation and return to Argentina afterwards. However, while packing up their luggage, Mrs. R left J’s favorite toy, his PlayStation, at his maternal grandparents’ house, and packed J’s clothes into a separate suitcase. As a consequence, J manifested an overt tantrum and started squealing loudly at the airport that he wanted to be protected from his father. Mr. R was shocked and ashamed of his child’s behavior and left his family at the airport. Following this incident, Mrs. R and J refused to speak or see Mr. R when he returned to Lebanon and accused Mr. R of physically and sexually assaulting J. Mr. R was not allowed to see his son through all the trials and tribulations.

### The mother’s point of view

During the interview, Mrs. R accused her husband of physically abusing her shortly after their wedding. She claimed that her husband lied about living with her in Lebanon, the reason why she was asking for divorce. In her allegations during the divorce process, Mrs. R did not mention any physical assault by her husband. On the contrary, she claimed that she stayed in touch with Mr. R for the sake of J until “her husband stopped calling her.” She admitted refusing to give Mr. R the right to see J alone, under the pretext of protecting her child from any harm/endangerment. She described their lives in Argentina as horrible and disclosed thoroughly the situation they were living in. She characterized Mr. R as a brutal man who was very aggressive with J.

According to her, Mr. R was beating J and molesting him by touching him on his breast area and licking his ears with excitement. She claimed that she could not protect herself or J because Mr. R did not let her interfere in the father–son relationship. As a result, she claimed that J refused any contact with Mr. R to keep himself and his mother safe from the relentless behavior of his father. She also attested that Mr. R would physically abuse J by hitting and spanking him violently. Consequently, J had pain in his hands and body, which prevented him from being able to write in school. She claimed that she took J once to the police station, where the police officers recommended arresting Mr. R. However, she refused to take further legal actions in the interest of protecting her family and because she was not willing to end up living in Argentina alone with no friends or family members.

She also reported that, following the child’s nervous breakdown at the airport, symptoms of fatigue, sadness, lack of concentration, social withdrawal, isolation, anhedonia, and unjustified fears began to appear. He was diagnosed later on with anxious–depressive disorder and was prescribed clomipramine 10 mg twice daily and diazepam 2.5 mg in the evening and 5 mg at bedtime. She claimed that the psychiatrist and psychotherapist reported physical violence but no sexual assault, and that J’s psychiatrist revealed in a private call with her that the child reported some sexual abuse by his father.

Mrs. R implored the sparing of her son of any questions and interviews concerning this matter, as J was too sensitive when asked about this story. J, who was present at the time of the interview with his mother, showed signs of anxiety, fatigue, and physical signs of nausea and abdominal pain. Mrs. R said that her son was not feeling any better, despite taking his psychiatric treatment, especially when he knew about the return of his father to Lebanon. He felt afraid and refused to leave his house, preferring to play computer games in his room. She added that J had thrown away his PlayStation, as it is a gift from his father. She also told us that J was willing to change his last name to his mother’s family name. In fact, Mrs. R presented her son with her family name instead of his father’s last name upon introducing J to us. She claimed that she was doing that to protect her son from his father, and that she is quite capable of raising her son with her parents’ help without any intervention from Mr. R.

### J’s story

J, the son of Mr. and Mrs. R, considered himself a particularly good student, having only a few difficulties in Arabic courses after returning to Lebanon from Argentina, since he did not take any Arabic courses in Argentina. In school, he preferred acting classes as an extracurricular activity. He claimed that he had made good friends with his maternal cousins and was happy living with his maternal grandparents. He said that he always considers his maternal grandfather to be his real father and Mr. R to be his uncle.

When speaking about his father, he stated weak, illogical reasons for his anger and fear towards his father. J was very confident about these negative feelings and confessed that he wanted to see his father dead. He even claimed that he would kill himself or his father if he was forced to see him.

He declared that no one instructed him about what to say, and he was only expressing his feelings about this matter. He said that he was physically assaulted by his father, and he was beaten up daily for no specific reason. He alleged that his father took him twice to strip shows in Argentina and forced him to watch pornography.

He described scenes where his father would abuse him physically and sexually every single day. When confronted with photos in which J appeared happy and smiling with his father, he said that he was obliged by his father to smile for the camera. He insisted that he was permanently sad in Argentina because of his father’s abuse.

J showed no regret or guilt towards his father but felt constantly guilty towards his mother because he was the one who accepted to go to Argentina and live with his father in the first place. He refused to see his father’s family because they were unpleasant towards him and reminded him of his father. J seemed fearful when speaking about his father and showed constant refusal to speak when asked about the whole story. He just wanted to forget his father and change his father’s family name to his mother’s. He showed some resistance and an overt refusal to visit his father, even when reassured that the visit would be supervised and safe.

## Discussion

This is the first reported case in Lebanon regarding a parental alienation case that occurred in the context of custody disputes during and after parental divorce, in which the mother intended to destroy the relationship of the child with his father by manipulating the child so that he would demonize his father. This paper focuses on the psychological motives behind the PA during divorce litigation of the alienated parent, the alienating parent, and the child, which seems to be important for optimal intervention for the child’s profit.

In opposition to the alienating parent, the alienated parent tends to describe their distress because their child is rejecting them. They usually describe how close their relationship was with the child and how distraught they felt when they attempted to approach or visit their son or daughter. Most targeted parents agree that their children should be raised in a loving environment, away from parent’s animosity and conflict. As a result, the targeted parents are usually not responsible for being rejected by the child, and therefore, do not usually exhibit any psychopathology [13].

However, some targeted parents describe alienating behavior as family violence, and negative automatic thoughts and feelings tend to be described by them [14]. In fact, about one-third of targeted parents report having attempted suicide [15]. However, in our case, the targeted parent tried to avoid being overly influenced by negative emotions by showing some control over them during interviews. This is a common active coping behavior style, which can be quite effective, especially if the person is flexible about its use, for a preservation of their self-image [16]. In fact, the father’s psychological assessment in our study showed that he is a very communicative and cooperative person. He was eloquent and direct in all his answers throughout the interviews and tended to describe events thoroughly. He stayed calm and self-controlled even when he was put in the accused position. He did not appear impulsive or carried away by his reactions or emotional expressions at any time. Ordinarily, he appeared to be intellectual, with preference to delay decisions or behaviors until he had considered various opinions. He strives to be logical and relies on his own internal evaluations rather than external feedback when making decisions. This strategy shows clear, reasonably sophisticated, and often creative thinking that reinforced the active role of the targeted parent to help his child’s independence and emancipation [17], as shown by the Rorschach test results in our study.

The alienating parent, however, usually justifies their doings by focusing on what is the best to be done in the interest of the child. Douglas Damal described such behavior in alienators as obsessions, where the alienating parent tends to rationalize their behavior by believing that the child is a victim of abuse [18] and, as a result, tends to destroy the relationship with the targeted parent. Such behavior usually relies on pathological personality traits or mental illness, showing a lack of personal self-control with a defensive attitude, which reinforces the delusion exhibited by the alienating parent of reasonably fearing and hating the nonpreferred parent [19]. In fact, the psychological assessment of Mrs. R showed an excessively inflexible, unrealistic, egocentric, self-centered attitude, with explicit contradictions in her ideas and expressions. Such a defensive attitude during clinical interviews creates doubts about the validity of her claims, not only among clinicians but also in court. In fact, courts had credited the mother’s claim of child abuse only one-third of the time [20]; child sexual abuse claims in custody litigation are rarely accepted when presented in courts compared with physical abuse claims alone [21]. Another study found that when allegations from the alienating parent are mixed, the odds of losing custody increase 2.5 times when claiming both sexual and physical abuse toward the child compared with sexual allegations only [8]. Our results were consistent with the results of this prior study, with no clear reasons behind this.

Mrs. R also lacks insight into herself and the ability to engage in self-criticism. She was completely and totally convinced that her allegations were justified and was totally committed to estranging her son from his abusive father. Obsessed alienators also tend to entrap their children’s beliefs into their own, projecting their own irrational delusions on the child and gaining a feeling of control of the situation [22]. In fact, Mrs. R has an excessively dependent and overprotective relationship with the child. The overall clinical and psychological assessment showed a paranoid personality disorder, frequently found among alienating parents [14, 23]. In addition, quenchless anger has been reported among obsessive alienators, who show poor coping skills and impulsive behavior, especially when put under stress [18]. The psychological testing of Mrs. R revealed some weakness in the control of affective and behavioral impulses, leading to expression of aggression and authoritarian behavior toward others. She lacked any impulse control and appeared very rigid and self-confident, features of grandiose and narcissistic behavior. She also tried to control the other parent [24]. Parents divorcing often realize that they still need to communicate with each other to ensure the child’s ordinary activities such as school functions, activities, and so on [25]. This is usually not the case in parental alienation, and the alienating parent does not believe anymore that they must communicate with the other parent for the best interest of the children, which could explain the narcissistic traits found in Mrs. R. In fact, she had found a way to express her anger through the child, using the child as a messenger to manifest feelings of revenge towards his father. As a matter of fact, Mrs. R’s psychological testing revealed her tendency to express aggressiveness without any significant provocation, with a restricted ability to show empathy and warm affect. Her inaptitude for self-criticism during interviews lead her to an explosive emotional outburst, making her look aggressive and unpleasant. One study found similar characteristics among alienated mothers, who show hostile impulses and unrestricted blame toward the targeted parent [26].

Since the alienated child was freely expressing hostile feelings toward the targeted parent, focusing on the psychological aspects of the child and his parental relationships could help the professional involved in the custody dispute to differentiate parental alienation from simple parenting divorce. After exhibiting refusal behavior in the first stage of custody dispute, J seemed to enjoy the company of his father while passing the holidays in Argentina. However, the relationship of J with his father strongly deteriorated after his return to the alienated parent, accusing his father of physically and sexually abusing him, even if the physical assessment of J did not show any sign of recent or old physical abuse. This is consistent with Joseph Goldberg’s assertion that the visitation process goes through three stages: resistance, pleasure, and confliction [27]. In fact, the alienating parent usually use strategies of threats and intimidation against the child whenever pleasure is felt during the visit of the alienated parent [28], removing by that any good feeling that could have arisen during the visitation’s pleasure phase. In doing so, the alienated child learns somehow to express rejection toward the alienated parent, avoiding the alienating parent’s negative reaction.

In addition, the alienated child usually lacks feelings of guilt and ambivalence towards the alienated parent [16] and finds it difficult to be separated from the alienating parent since the rejected parent is slowly being pushed out of his parental role. In fact, the psychological assessment of J showed a symbiotic relationship with his mother. He is bonding with her and finds himself unable to be separated from his mother. Thus, J, who was totally relying on his mother to have his needs met, could not develop a separate identity from her. J continued to be dependent on his mother, which encouraged the development of a complex mother-to-son relationship that motivated the denigration of his relationship with his father, by exhibiting his severe oppositional behavior toward him.

The symbiotic bonding of the child with the alienating mother is sometimes described as a Stockholm syndrome [29]. Imitating the alienating parent is one method used by the alienated child to survive the alienating parent’s psychological pressure, to regain their worthiness in the eyes of the alienating parent. Thus, maintenance of the negative emotional climate when around the alienated parent is essential to gain the trust of the alienating parent. Soon, the child will generalize their negative emotions to anything that relates to the alienated parent. In fact, such defamation leads to the loss of the children’s loving care and attention from the targeted parent, resulting in behavioral problems including attention difficulties, anxiety, depression, and aggression [30], and experiencing substantial losses across their lives later [31]. Studies show that adults who were child victims of parental alienation report low self-esteem, mood disorders, attachment, and interpersonal relationship problems [32–34]. They also tend to describe experiencing abuse by the alienating parent [34]. In our case, J had a nervous breakdown at the airport and was treated with antidepressants and anxiolytics after his psychiatric consultation, which shows the possibility of developing a mood disorder in adulthood.

## Conclusion

The data from the case study appear to be in line with Gardner’s observations of PA [10]. The mother turned the child against his father through powerful emotional manipulation techniques designed to bond with the child at the expense of excluding his father. The mother–son relationship did not encourage independent thinking skills in the child but rather motivated an unhealthy dependence toward her instead, satisfying through these means the emotional needs of the mother rather than the developmental needs of the child. The evaluation concluded that the mother’s inability to differentiate her own needs from those of the child led the child to target his father, characteristics found and confirmed in parental alienation.

## Data Availability

Not applicable.

## Abbreviations

PA: Parental alienation
PAS: Parental alienation syndrome
